# In Memoriam—Dr. James Kitchen

**Published:** 1894-09

**Authors:** 


### IN MEMORIAM—DR. JAMES KITCHEN.

Dr. James Kitchen, the oldest physician in this country or in the world, perhaps, died on Sunday evening, August 19th, 1894, at his home, No. 715 Spruce Street, Philadelphia. He was born March 8th, 1800, in Philadelphia, and received his classical education in the literary department of the University of Pennsylvania. At the same institution he studied medicine under Professor Thomas H. Hewson, and received his diploma in April, 1822. He then spent two years in the Paris hospitals and returning to this country in 1824, hung out his sign at No. 37 Spruce Street, Philadelphia. For fifteen years he conformed to the rules governing the practice of allopathy. At the expiration of that time he renounced his faith and launched forth into Homœopathy.

In this school he made for himself a reputation not restricted to the limits of Philadelphia.

Up until within a very short time of his death he continued the routine of his daily visits, and was hale and hearty; but a long time ago he found it necessary to discontinue night visits. The house in which he died is quaint and old-fashioned. It is a double one, and has stood tempest and sunshine for well on to a century.

Dr. Kitchen's father, James Kitchen, came to Philadelphia from Wales in 1790, and was the proprietor of a famous resort in the latter part of the last century, and early in this, known as the Merchants' Exchange.

The young James was born in a house on what is now known as Second Street, near Walnut, Philadelphia. Dr. Kitchen was a man of average height. His eyes were bright, but his long white hair and beard gave him a very venerable appearance. He enjoyed remarkable health; but when the grippe swept over the country he fell a victim, and his hearing and sight were slightly affected by it, though even after that he could read ordinary print without the use of glasses.

In 1891, Dr. Kitchen in a conversation gave some reminiscences:

"When I was born," said the Doctor, "Philadelphia was a town of 70,000 people, and now I have seen an increase of over a million. This house where I now live I first moved into in 1853. It was built in 1828 by a Canton merchant named Whitney, who spent two years in completing it. At that time that ground on the other side of the street (now occupied by a row of three-story brick houses) was a cornfield, inclosed by an ordinary rail fence.

"Whitney broke and never occupied the house, spending the latter years of his life in an insane asylum. In those days Philadelphia monopolized the trade of America, and here lived many wealthy merchants who carried on trade with foreign nations. Whitney owned a number of sailing vessels which traded at Canton, China, and East India ports. Every spring the Philadelphia merchants had auction sales, the amount realized running from $10,000,000 to $20,000,000.

"About 1828, however, the craze for internal improvements set in, and the Pennsylvania Legislature determined to construct a number of canals. The question, of course, arose as to how the money was to be raised, and it was decided to levy a tax of $1 on every $100 worth of goods imported into Philadelphia. The canals were constructed, but many merchants were broken, and trade at once began to drift to New York, Boston, and other ports.

"There has been a wonderful change in Philadelphia since my boyhood days. All of my early friends are dead. None remain at all, with the exception of my sister, who lives with me, and who is eighty-seven years of age." This sister died about a year ago.

The Doctor never married, giving as an excuse for not joining the ranks of the Benedicts that in his experience as a physician he saw so much family trouble that he determined many years ago to remain single. Besides his sister he had a niece who lived with him, and her children made the house lively and recalled to him the days, over three-quarters of a century ago, when he ran and romped with his juvenile friends in the infancy of the Republic.

In a letter to the editor of this journal Mrs. Maria Cowell, the niece referred to, said of him: "For fifteen years he practiced allopathy, but having some chronic liver trouble, which had been pronounced incurable, he was persuaded by his old boyhood's friend, Dr. W. T. Helmuth, to try Homœopathy, with the promise if it cured he would look into that school. He was cured, kept his promise, and became a convert, and never lost his faith in or love for old Hahnemann's principles to the last day of his life. He was one of the brightest of companions, never low-spirited, always taking things as they came, and thinking all one did for him was right. I never heard him speak a cross word. He was very fond of children, and a great tease, always ready to enter into any fun or anything that would give pleasure to others. He was very generous. There is many a home that will miss him, for he was always doing good in a quiet way of his own. He had such merry blue eyes, they would sometimes be just brimming over with fun. He scarcely ever laughed aloud, just a low h-m. When he did laugh out, it was so contagious everybody near him had to laugh also. The family called him their encyclopædia, because, no matter what subject they needed information on, he could tell them just what they wanted to know. He wrote a great number of articles for the medical magazines and translated several works, but his life work is done now, and done well, and the dear Lord has taken him home, where there will be no more pain, only everlasting joy. If he only could have done as Elijah did, leave his mantle, or, rather, a little of his knowledge behind for some of us who are left, how thankful I would be. It does seem as though it ought not all to be lost."

